# Sympathetic Shift and Insular Alteration: Unravelling the Link Between Anxiety and Heart Rate Variability in Parkinson's Disease

**DOI:** 10.1002/mds.70069

**Published:** 2025-10-07

**Authors:** Lucia Ricciardi, Alessandra Fanciulli, Francescopaolo P. Cucinotta, Bryony Ishihara, Ioana Cociasu, Fahd Baig, Michael Hart, Erlick Pereira, Francesca Morgante, Elena Makovac

**Affiliations:** ^1^ Neurosciences and Cell Biology Institute, Neuromodulation and Motor Control Section, City St George's University of London London UK; ^2^ Dysautonomia Center, Division of Clinical Neurobiology, Department of Neurology Medical University of Innsbruck Innsbruck Austria; ^3^ Centre for Neuroimaging Science, King's College London UK; ^4^ Brunel University London Uxbridge UK

**Keywords:** anxiety, autonomic dysfunction, heart rate variability, insula, Parkinson's disease

## Abstract

**Background:**

Anxiety and autonomic dysfunction are frequent non‐motor symptoms of Parkinson's disease (PD). Their relationship, as well as the neural mechanisms underlying this relationship, remain unexplored.

**Objectives:**

We aimed to investigate the relationship between cardiovascular functions and anxiety in PD and the structural neural changes underlying this putative interaction. We also investigated the effect of dopaminergic medications on such a relationship.

**Methods:**

Fifty PD patients (27 with anxiety, PD_Anx; 23 without anxiety, PD_noAnx) and 16 age‐ and gender‐matched healthy controls (HC) were included. Blood pressure (BP), heart rate, and heart rate variability (HRV) were assessed at rest and in response to an orthostatic challenge, both ON and OFF dopaminergic medications. Voxel‐based morphometry was used to examine grey matter volume in brain areas linked to autonomic regulation and anxiety, including the amygdala, insula, cingulate cortex, hypothalamus, and putamen.

**Results:**

PD_Anx patients showed significantly lower HRV compared with both PD_noAnx patients and HC (*P* < 0.05), indicating increased sympathetic activity. Both PD groups had higher BP OFF medication compared with HC (*P* < 0.001, *P* < 0.005, respectively); there was no difference between PD_Anx and PD_noAnx (*P* = 0.31). Structural brain analyses showed that anxiety altered the relationship between HRV and left insula volume, with a positive correlation in PD_noAnx patients and a reversed relationship in PD_Anx patients.

**Conclusions:**

Anxiety in PD is associated with a shift toward sympathetic predominance, which correlates with structural changes in the insula. Insular alteration may predispose PD patients to heightened sympathetic outflow and anxiety. Changes in HRV may be interpreted as a functional indicator of anxious states in PD. © 2025 The Author(s). *Movement Disorders* published by Wiley Periodicals LLC on behalf of International Parkinson and Movement Disorder Society.

In Parkinson's disease (PD) non‐motor symptoms, such as neuropsychiatric and autonomic dysfunction, are very common and significantly impact patients' quality of life.^1^ Among the neuropsychiatric symptoms, anxiety is one of the most disabling, often appearing up to 20 years before motor symptoms.^1^ Its prevalence ranges from 6% to 55%, averaging 31%,^2^ much higher than the general population (9.8%).^3^ The mechanisms behind anxiety in PD are unclear, and effective treatments are lacking.

Autonomic dysfunction is also highly prevalent in PD, often involving multiple autonomic domains at the same time, and presenting with urinary urge, constipation, heat intolerance, sweating abnormalities, and orthostatic intolerance. Nearly all PD patients experience some degree of autonomic dysfunction at some stage of the disease, significantly contributing to its overall burden.^4^

Studies, based on self‐report questionnaires, suggest a link between autonomic dysfunction and anxiety in PD, with cardiovascular and thermoregulatory issues predicting the future development of anxiety.^5^, ^6^, ^7^ The relationship between autonomic dysfunction and anxiety in PD is complex, potentially involving both peripheral and central mechanisms. Peripheral autonomic changes, such as sympathetic cardiac denervation, have been linked to anxiety in PD.^8^

Heart rate variability (HRV), the variation in the time interval between heartbeats, is a quantitative measure of autonomic regulation, particularly vagal modulation.^9^ HRV is linked to physical and mental well‐being^10^, ^11^ and is associated with psychological and physiological flexibility, making it a potential biomarker for stress resilience.^12^ Indeed, lower HRV, indicating impaired cardiovagal outflow to the heart, has been shown in affective disorders such as depression and anxiety.^13^, ^14^, ^15^ Recent meta‐analyses^16^, ^17^ show that PD patients generally have lower HRV than healthy controls (HC), though there is significant inconsistency in the findings, with some studies reporting no difference between the two groups.^16^ Dopaminergic medications might be another factor influencing HRV in PD and the heterogeneity of these results, but to date the effect of medications on HRV remains unclear.^16^, ^18^, ^19^

Neuroimaging studies in PD have shown alterations in structures of the central autonomic network,^20^, ^21^ including the prefrontal cortex, cingulate cortex, insula, and brainstem.^22^, ^23^ Changes in these areas are also implicated in anxiety in PD,^24^ suggesting a potential mechanism connecting anxiety and central autonomic network changes in PD.

No study to date has examined objective measures of cardiovascular autonomic function in relation to anxiety in PD patients, and the neural mechanisms underlying this putative interaction.

This study aimed to investigate this relationship using objective physiological measures in PD patients with and without anxiety (PD_Anx, PD_noAnx) and age‐ and gender‐matched HC, evaluating HRV and BP at rest and in response to an orthostatic challenge.

To explore the effect of levodopa on the relationship between anxiety and cardiovascular reactivity, we assessed PD patients both ON and OFF their regular medications.

Voxel‐based morphometry (VBM) was used to examine brain structural differences between PD_Anx and PD_noAnx, and the association between these regions and HRV and BP in both PD groups.

Our hypotheses were:

1. PD_Anx have reduced HRV compared with PD_noAnx and to HC.
2. Structural brain differences are observed in the regions of the central autonomic network between PD_Anx and PD_noAnx.
3. The relationship between HRV and brain volume is moderated by anxiety status.^20^, ^21^

## Methods

PD patients were recruited from the movement disorders clinic at St George's University Hospital in London, UK. HC were recruited among volunteers and clinical/research staff. All participants provided written informed consent under the Declaration of the Principles of Helsinki. Approval was obtained from the Institutional Review Board of the Integrated Research Application System. Inclusion criteria were: age > 18 years; ability to give informed consent; for the patients a diagnosis of PD according to the MDS criteria^25^; for the HC, absence of anxiety and/or depression as per clinical interview. Exclusion criteria were presence of any neurological and psychiatric disorder, including dementia as per clinical diagnosis and a Montreal Cognitive Assessment score ≤ 21; cardiovascular or any other medical condition or medication interfering with heart rate (eg, beta‐blocker); and uncontrolled diabetes and other causes of autonomic neuropathy.

Participants were assessed in the morning, OFF (12 h after medication withdrawal) and ON (1 h after levodopa) medication. The PD protocol included clinical evaluation, physiological assessment, and questionnaires, while the HC protocol included an interview to exclude anxiety and depression (as per inclusion criteria) and the same physiological assessment.

### Clinical Evaluation

In HC and PD patients we collected demographic data (gender, age), medical history, current medications, lifestyle factors (nicotine, alcohol, caffeine intake, physical activity), and the use of antidepressants and anxiolytics. In patients only we recorded disease duration and PD symptoms. Participants were instructed to avoid caffeine, alcohol, and nicotine on the examination day.

PD patients underwent a clinical examination evaluating:

- Motor symptoms: Unified Parkinson's Disease Rating Scale (UPDRS) Part III.
- Non‐motor symptoms: clinical interview plus the Hamilton Anxiety Rating Scale (HARS) and the Hamilton Depression Rating Scale (HAM‐D), the Scales for Outcomes in Parkinson's Disease‐Autonomic Dysfunction (SCOPA‐AUT).^26^

Anxiety classification was informed by Diagnostic and Statistical Manual of Mental Disorders, Fifth Edition (DSM‐5) criteria and based on the presence of anxiety symptoms identified during clinical interviews conducted by an experienced neurologist and neuropsychologist, supported by elevated scores on the HARS. Accordingly, participants were categorized as PD_Anx or PD_noAnx.

### HRV and Blood Pressure Assessment

Systolic and diastolic blood pressure (BP) were measured at rest (sitting) and 1 and 3 min after standing using an arm cuff.^27^ Participants rested in a sitting position for 5 min before continuous HRV data recording for 3 min, followed by 3 min of standing while HRV was recorded. Beat‐to‐beat cardiac intervals (RR) were captured using a Polar H10/H7 chest band (Polar Oy, Finland), validated for accuracy,^28^ and sent via Bluetooth to the HRV Logger app (iOS) for recording. Orthostatic hypotension was defined as a ≥ 20 mmHg drop in systolic or ≥ 10 mmHg drop in diastolic BP within 3 min of standing.^29^

### HRV Preprocessing

HRV data were extracted and the root mean square of successive differences between normal heartbeats (RMSSD‐an index of vagally‐mediated HRV^30^) was calculated with Kubios^31^ for epochs of 1 min while sitting and standing. Artefacts and ectopic beats were corrected using a threshold‐based correction.

HRV variables were logarithmically transformed. logRMSSD in sitting (at minute 3, following a 2 min stabilization period), and after 1 min, and 3 min upon standing were used in the ANOVAs to characterize dynamic control adjustments.^32^

Delta_logRMSSD values were calculated by subtracting the logRMSSD measured while standing from the logRMSSD measured while sitting at both the first and third minute, and for conditions when patients were ON and OFF medication (Delta_logRMSSD). Negative Delta_logRMSSD values indicated lower logRMSSD while standing compared with sitting, suggesting a decrease. Conversely, positive values indicated an increase. A decrease in logRMSSD indicates reduced parasympathetic (vagal) activity and a shift towards increased sympathetic activity.^33^

### MRI Acquisition and Preprocessing

MRI acquisitions were performed only in PD patients, who were scanned on a separate day from the clinical assessment using a 3.0 Tesla (3 T) scanner while ON medication. Structural volumes were obtained using a high‐resolution T1‐weighted MPRAGE sequence, and brain extraction was conducted using the FSL BET tool.^34^ Preprocessing followed established protocols, including normalization, segmentation, and smoothing procedures performed in SPM12 (Statistical Parametric Mapping, http://www.fil.ion.ucl.ac.uk/spm/) using the DARTEL algorithm. Full methodological details are provided in the Supplementary Material.

### Statistical Analysis

Demographic and clinical data were compared using *t*‐test, Mann–Whitney U test, or chi‐square test as appropriate and according to data distribution. A post‐hoc sample size calculation is presented in the Supplementary Material.

To test hypothesis 1, a 3 × 3 ANOVA was conducted for each dependent variable, logRMSSD and BP, with Group as between‐group factor (HC, PD_Anx, PD_noAnx) and Position (sitting, standing minute 1, standing minute 3) as within‐group factors. These analyses were performed separately for the ON and OFF medication conditions.

To test the effect of levodopa in the PD group, we then ran repeated‐measure ANOVA with *Medication* (ON, OFF) and *Position* (sitting, minute 1, minute 2) as within‐group factors, and *Group* (PD_Anx, PD_noAnx) as a between‐group factor for logRMSSD and BP.

Where significant main effects or interactions were observed, post‐hoc pairwise comparisons were conducted using *t*‐tests.

Pearson correlations were performed across the PD_Anx and PD_noAnx groups to examine associations between HRV and demographic/clinical variables.

A Bonferroni correction was applied to adjust for multiple comparisons. The significance threshold for each test was set at *α* = 0.0125, calculated by dividing the conventional alpha level of 0.05 by the number of correlations between our primary variables of interest: HARS and the four logRMSSD conditions of interest (ON minute 1, ON minute 3, OFF minute 1, OFF minute 3).

Differences at *P* < 0.05 were regarded as significant. Data analysis was performed with SPSS 23.0 (SPSS Inc., Chicago, IL, USA).

VBM Analyses (to test hypotheses 2 and 3): Statistical analyses were conducted on smoothed grey matter (GM) maps within the framework of the general linear model. For VBM analyses, we aimed to identify brain regions that were positively or negatively associated with logRMSSD and BP measures, either at rest or in response to orthostatic change (Delta_logRMSSD and Delta_SysBP), ON and OFF medication. Physiological and clinical scores (HARS) were treated as variables of interest in the regression analyses, while intracranial volume was included as a nuisance covariate to account for potential confounding effects due to individual differences in head size.

Statistical thresholds were set to *P* < 0.05–family‐wise error (few)‐corrected at cluster level^35^ (cluster size defined using uncorrected voxel‐level threshold *P* < 0.001). Total intracranial volume, age, gender and disease duration, and total LEDD were included as covariates of no interest in subsequent general linear model analyses.

## Results

Fifty PD patients (27 PD_Anx, 23 PD_noAnx) and 16 HC were included in the study. Static heart rate was analyzed in 22 PD_noAnx patients, as data for 1 patient was missing. At minute 3 (OFF, standing), HRV data were missing for 1 HC, 3 PD_Anx, and 3 PD_noAnx, so analyses used 15 HC, 20 PD_noAnx, and 24 PD_Anx.

There was no difference between the PD_Anx and PD_noAnx in any demographic (age, biological sex) or clinical variables (disease duration, UPDRS‐III OFF and ON medication, levodopa equivalent daily dose [LEDD] dopamine agonists) except for total LEDD, where PD_Anx had higher dose than PD_noAnx (*P* = 0.04) (Table S1).

Thirty‐two PD patients were on dopamine agonists. Thirty‐four used catechol‐O‐methyltransferase (COMT)/monoamine oxidase B (MAO‐B) inhibitors (13 PD_noAnx, 21 PD_Anx, *χ* ^2^ = 1.69, *P* = 0.2) and 10 selective serotonin reuptake inhibitors (SSRIs)/anxiolytics (2 PD_noAnx, 8 PD_Anx, *χ* ^2^ = 2.22, *P* = 0.136). Five PD_noAnx and 3 PD_Anx met the criteria for orthostatic hypotension OFF medication, while 6 PD_noAnx and 4 PD_Anx did so ON medication.

PD_Anx reported worse symptoms of thermoregulatory dysfunction (excessive perspiration and heat/cold intolerance) than PD_noAnx as per SCOPA Thermoregulatory subscore (*P* = 0.03) (Table S1).

### Comparison Between PD Patients and HC

#### HRV

When testing PD subjects OFF medication in comparison with HC, we observed a main effect of the factors Position (*F*(2, 55) = 11.69, *P* < 0.001) and Group (*F*(1, 55) = 21.757, *P* = 0.004). We did not find any Position*Group Interaction (*F*(4, 55) = 1.06, *P* = 0.38). The main effect of Position was driven by a decrease in logRMSSD from sitting to standing at both minute 1 and minute 3 (*P* = 0.031 and *P* < 0.001). LogRMSSD was also lower in standing minute 3 versus minute 1 (*P* = 0.01).

**FIG. 1:**
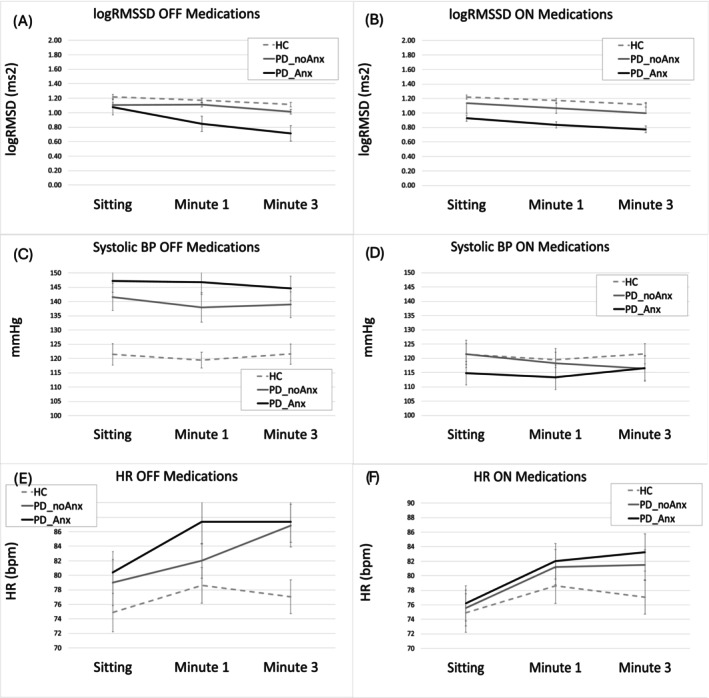
Effect of position on heart rate variability (logRMSSD) (A, B), blood pressure (C, D), and heart rate (E, F) in healthy controls (HC), patients with anxiety (PD_Anx), and patients without anxiety (PD_noAnx), separately for the ON and OFF position.

The main effect of the factor Group was driven by an overall lower logRMSSD in PD_Anx versus HC (*P* = 0.04). No difference was observed between PD_Anx and PD_noAnx or between PD_noAnx and HC (*P* = 0.28 and *P* = 0.25, respectively) (Fig. 1A).

When testing PD subjects ON medication in comparison with HC, we observed a main effect of the factors Position (*F*(2, 55) = 10.45, *P* < 0.001) and Group (*F*(1, 56) = 6.67, *P* = 0.002). No effect of the Position*Group Interaction (*F* < 1). The main effect of Position was driven by an overall decrease in logRMSSD from sitting to standing position at both minute 1 and minute 3 (*P* = 0.008 and *P* < 0.001). The main effect of the factor group was driven by an overall lower logRMSSD in PD_Anx versus HC (*P* < 0.001), and between PD_Anx and PD_noAnx (*P* = 0.035) (Fig. 1B).

#### Blood Pressure

OFF medication, we observed a main effect of the factor Group (*F*(2, 52) = 8.74, *P* < 0.001). The factor Position (*F*(1, 52) = 1.47, *P* = 0.23) or the Position*Group Interaction had no effect (*F* < 1). The main effect of the factor Group was driven by an overall higher systolic BP in PD_Anx and PD_noAnx as compared with HC (*P* < 0.001; *P* < 0.005); there was no difference between PD_Anx and PD_noAnx (*P* = 0.31) (Fig. 1C).

ON medication, there was no significant effect of the factors Group (*F* < 1), Position (*F* < 1), or Position*Group (*F* < 1, Fig. 1D).

### Dopaminergic Medications Effect on Autonomic Measures in the PD Populations

logRMSSD (Fig. 1A,B): To directly compare the effect of levodopa and anxiety on logRMSSD, we computed a 2 × 3 × 2 ANOVA with Group as between‐groups factor (PD_Anx, PD_noAnx) and Position (sitting, standing minute 1, and minute 3) and levodopa (OFF, ON) as within‐group factors, controlling for disease duration and age. There was a main effect of Group (*F*(1, 36) = 7.93, *P* = 0.008), with PD_Anx showing lower logRMSSD than PD_noAnx (0.89 ± 0.29 vs. 1.05 ± 0.32), but no difference between PD_noAnx and HC. No significant effects were found for Position, Medication, or any interaction (*F* < 1). The results were maintained when controlling for LEDD.

SysBP (Fig. 1C,D): A 2 × 3 × 2 ANOVA with Group as between‐groups factor (PD_Anx, PD_noAnx) and Position (sitting, standing minute 1, and minute 3) and levodopa (OFF, ON) as within‐group factors, controlling for disease duration and age, showed no significant main factors (Medication and Group [*F* < 1], Position [*F*(2, 30) = 1.44, *P* = 0.25]).

There were no significant interactions between factors: Medication*Position (*F*(2, 30) = 1.36, *P* = 0.29), Medication*Group (*F*(1, 30) = 2.89, *P* = 0.10), Position*Group (*F* < 1), and Medication*Position*Group (*F* < 1). The results were maintained when controlling for LEDD.

### Correlation Analysis in PD Patients' Group

**FIG. 2:**
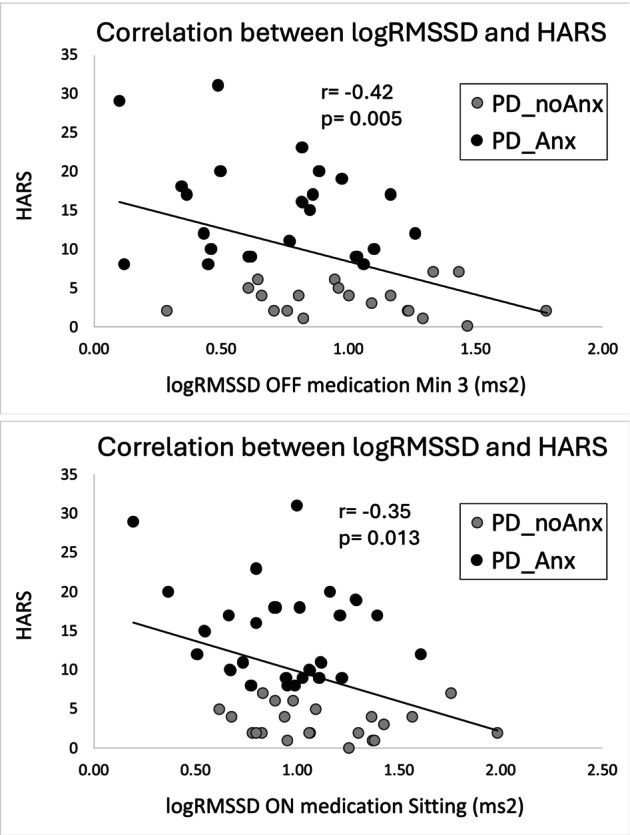
Correlations across both groups of patients with anxiety (PD_Anx) and patients without anxiety (PD_noAnx) between Hamilton Anxiety Rating Scale (HARS) scores and Delta_logRMSSD. Upper panel: Delta_logRMSSD at minute 3, OFF medications. Lower panel: Delta_logRMSSD in sitting position, ON medications.

In the whole patients group there was a significant negative correlation between anxiety (HARS) and logRMSSD OFF medication standing at minute 3 (*r* = –0.42, *P* = 0.005) (Fig. 2). This correlation was maintained when controlling for age, disease duration, and total LEDD (*r* = −0.45, *P* = 0.004). Both correlations remained significant after Bonferroni correction. A negative correlation was observed between HARS and logRMSSD ON medication sitting (*r* = −0.35, *P* = 0.013), even after correcting for disease duration, age, and total LEDD (*r* = −0.29, *P* = 0.039). However, these correlations did not survive Bonferroni correction.

### VBM

#### GM Differences Between PD_Anx and PD_noAnx

**FIG. 3:**
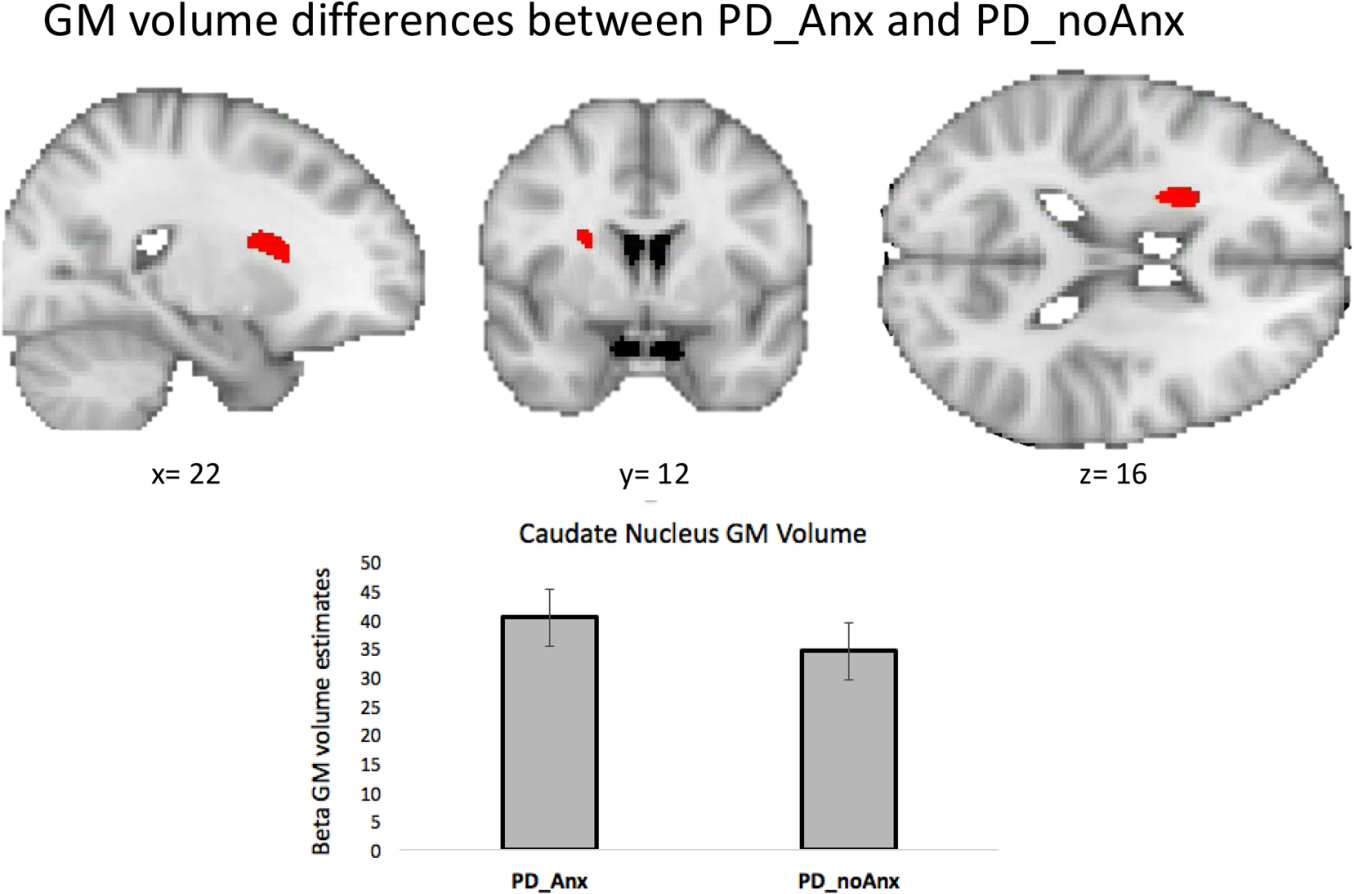
Difference in grey matter (GM) volume of the right caudate nucleus between patients with anxiety (PD_Anx) and patients without anxiety (PD_noAnx). GM volume parameter estimates were extracted from significant voxels and plotted for illustrative purposes. [Color figure can be viewed at wileyonlinelibrary.com]

When comparing grey matter (GM) volume between PD_Anx and PD_noAnx, higher GM volume was observed in the right caudate nucleus in the PD_Anx group (Fig. 3). No areas of decreased GM volume were observed comparing PD_Anx with PD_noAnx.

#### Correlational Analyses Between GM Volume and Autonomic Measures

**FIG. 4:**
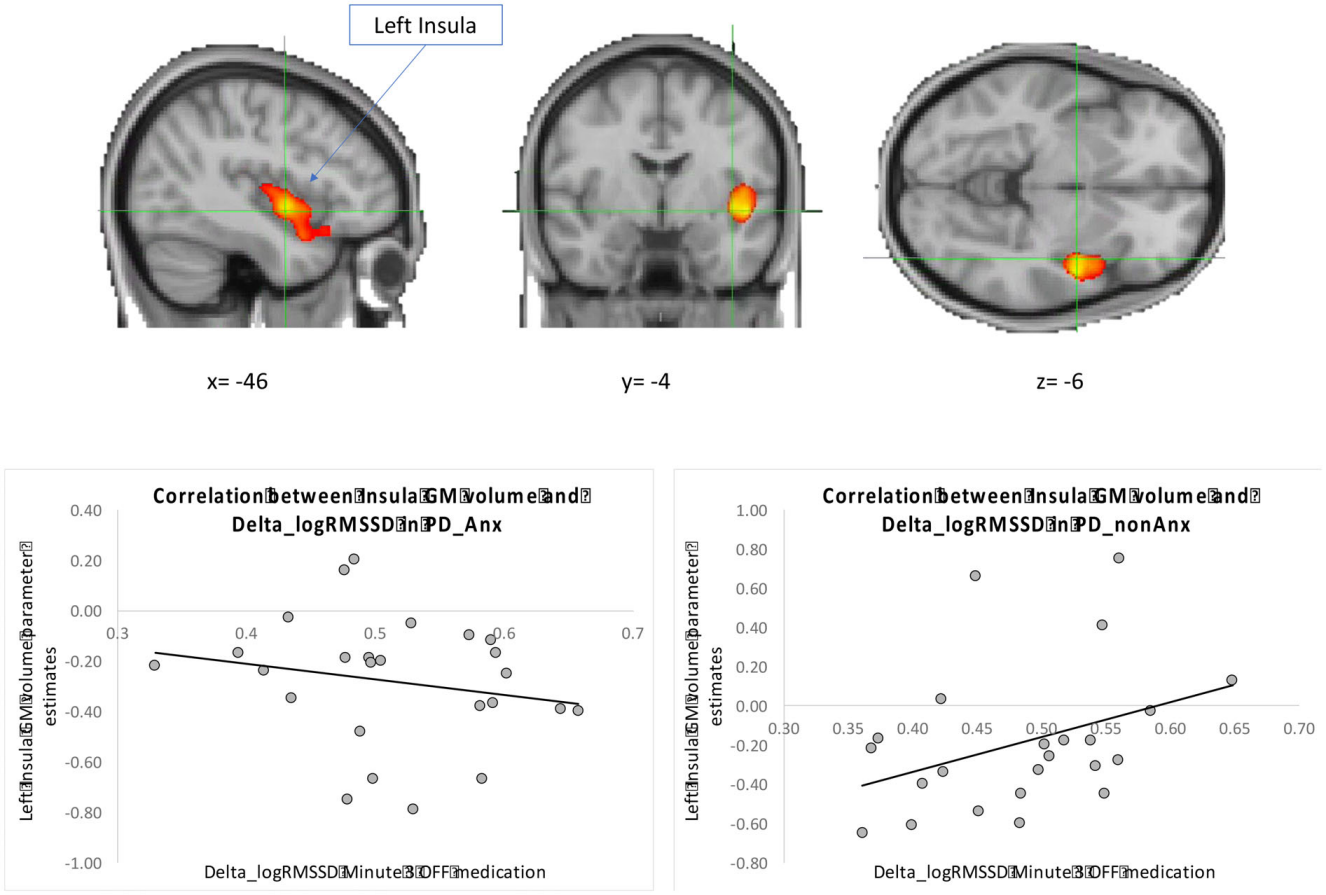
Differential correlation between Delta_logRMSSD and grey matter (GM) volume left insula cortex between the patients with anxiety (PD_Anx) and patients without anxiety (PD_noAnx) groups. In the PD_noAnx group, a positive correlation indicates that higher logRMSSD in the standing position compared with sitting is associated with increased GM volume in these regions. Conversely, in the PD_Anx group, this association was disrupted. GM volume parameter estimates were extracted from significant voxels and plotted for illustrative purposes. [Color figure can be viewed at wileyonlinelibrary.com]

The relationship between left insula GM volume and Delta_logRMSSD varied between PD_Anx and PD_noAnx (Fig. 4; Table 1). In PD_noAnx, there was a positive correlation where higher Delta_logRMSSD OFF medication at minute 3 was associated with greater left insula GM volumes. Conversely, in PD_Anx, this relationship was disrupted and did not reach statistical significance.

**TABLE 1:** Differences in grey matter (GM) volume between patients with anxiety (PD_Anx) and patients without anxiety (PD_noAnx) and correlation with Delta_logRMSSD

					Voxel			
Contrast	Brain area	Cluster/peak level^a^		Side	*T* (F)	MNI		
		*k*	*P*‐FWE			*x*	*y*	*z*
Group difference in GM volume								
*PD_Anx > PD_noAnx*								
Caudate nucleus^b^		54	0.029	Right	4.21	22	12	15
Correlation with logRMSSD								
Group × Delta_logRMSSD interaction								
Planum polare/insular cortex		1928	0.000	Left	5.95	−46	−4	−6
Frontal orbital cortex					3.67	−40	18	−16

*Note*: *P*‐FWE; *P*‐value adjusted for family‐wise error (FWE) correction: This *P*‐value has been adjusted to account for FWE, which corrects for multiple comparisons across numerous voxels. MNI: Montreal Neurological Institute coordinates. *k*: Represents the total number of voxels included within a statistically significant cluster. MNI*xyz*: These specify the location of the most significant voxel within the cluster based on the standard MNI system.

^a^ Peak level.

^b^ Small volume corrected.

## Discussion

In this study we investigated the relationship between cardiovascular regulation and anxiety in PD using physiological objective measures, namely RMSSD (a measure of vagally‐mediated HRV) and BP during an orthostatic challenge, and the neural basis of this interaction.

### Relationship Between Anxiety and HRV

Our results show lower HRV in PD_Anx compared with both HC and PD_noAnx. A reduction in HRV in PD as compared with HC has been reported previously^16^, ^17^ reflecting autonomic dysfunction due to degeneration in the autonomic nervous system both at the peripheral and central level.^36^ The novelty of our study lies in the finding that the reduction in HRV seems to be driven by PD_Anx, independent of disease duration, age, or dopaminergic medication load, highlighting a mechanism that links anxiety with autonomic functioning in PD. Anxiety is known to affect the autonomic nervous system, driving the shift towards sympathetic predominance and potentially leading to further dysregulation of heart rate control.^14^ Consistently, lower HRV has been shown in several neuropsychiatric conditions.^37^, ^38^ This is further supported by our correlation analysis showing a negative association between anxiety and HRV at rest ON medication, and the HRV OFF medication standing. An alternative explanation is that a diminished/impaired parasympathetic (cardiovagal) outflow facilitates the development of anxious symptoms in PD. To unravel the complex causal relationships between these variables, further longitudinal studies are necessary.

Interestingly, the reduction of HRV in PD_Anx appeared more pronounced when patients were ON medication. However, when this hypothesis was directly tested using medication as a factor of interest in the PD cohort, no significant interaction between anxiety and medication was observed. Unfortunately, definitive conclusions cannot be driven, as the lack of interaction may be attributed to low statistical power in our sample. The role of levodopa on HRV remains unclear, results from few previous studies are inconsistent, with some reporting reduced HRV in PD patients on chronic dopaminergic treatment,^16^ while others showed an increase in HRV following a single dose of levodopa, particularly within the first hour,^18^, ^19^ potentially accounting for the known hypotensive effects of dopaminergic medications.

### Blood Pressure and Heart Rate Findings: Role of the Baroreflex

When OFF medication, both PD groups (with and without anxiety) had overall higher BP and a greater increase in heart rate upon standing compared with HC. This agrees with previous research showing that hypertension and fluctuating BP are common in PD patients evaluated with a 24‐h monitor,^39^, ^40^ as well as with other studies showing a dysregulation of the baroreflex in PD.^41^

When ON medication, PD groups did not differ from HC in terms of BP and heart rate. This aligns with previous studies showing that levodopa administration decreases BP in PD patients but does not necessarily produce greater orthostatic BP falls.^42^ Interestingly, dopaminergic medications load (LEDD) was associated with both BP and heart rate when ON medication, with increased dosage associated with increased heart rate and decreased BP (indicating a baroreflex compensation of the lower BP with an increase in heart rate), confirming the interaction between dopaminergic medications and the baroreflex control in PD patients.

Interestingly, no significant BP differences were observed between PD_Anx and PD_noAnx across conditions (ON/OFF medication, sitting/standing), suggesting that anxiety may not substantially impact BP regulation in PD. This contrasts with our findings on HRV, indicating that while anxiety is associated with HRV, its relationship with BP may involve overlapping but distinct autonomic mechanisms in PD, pointing to HRV as a functional indicator of the anxious state in PD. While both BP and HRV are under baroreflex control,^43^ they provide complementary yet distinct information. HRV offers the advantage of reflecting dynamic autonomic responsiveness and adaptability to stress, while BP primarily provides a static measure of cardiovascular load.

Our data reveal a distinct pathophysiological mechanism in PD linking anxiety and autonomic dysfunction, confirmed through rigorous physiological measures, clinical interviews, and questionnaires. This approach addresses limitations in prior studies that suggested an association between anxiety and dysautonomia in PD^5^, ^6^, ^7^, ^44^ relying only on self‐reported scales. Our findings of sympathetic predominance in PD_Anx align with recent reviews suggesting a noradrenergic PD subtype,^45^ characterized by early dysfunction in central and peripheral noradrenergic transmission, leading to non‐motor symptoms such as anxiety and dysautonomia.

### Structural Brain Correlates of Anxiety in PD

We found increased GM volume in the right caudate nucleus in the PD_Anx group compared with PD_noAnx. Previous studies have linked anxiety in PD to the right caudate, showing a negative correlation between anxiety severity and dopamine transporter (DAT) availability,^46^ while others report higher DAT binding in PD patients with affective symptoms in the bilateral caudate and putamen.^47^ Hypertrophy of the striatum, including the caudate nucleus, has been described in anxious clinical populations.^48^ A hypertrophic caudate nucleus has also been linked to excessive daytime sleepiness in PD,^49^ a symptom that has been associated with autonomic dysfunction and anxiety in longitudinal studies,^50^ pointing to a potential role of this structure in characterizing different PD clinical phenotypes.^45^

A positive correlation was observed between changes in HRV and left insula GM volume when OFF medication, in PD_noAnx. This suggests that higher HRV in the standing position is associated with greater left insular volume. Whereas, in PD_Anx, this relationship was reversed or disrupted, indicating that anxiety alters or impairs the typical interaction between HRV and left insula volume. Anxiety may predispose individuals to a chronic autonomic shift toward sympathetic predominance, affecting insula structure. Alternatively, changes in the insula due to PD could drive a shift towards a sympathetic predominance of cardiovascular autonomic control predisposing some patients to experience anxious symptoms. In line with our results, earlier studies in people with generalized anxiety disorder showed that lower insula volumes predict stronger decrease in HRV in response to a worry induction.^51^

Our results emphasize the insula's central role in autonomic regulation.^52^ Insula is also a key hub for interoception—the ability to sense internal bodily signals, which regulates emotions and behavior.^53^ Altered interoception has been linked to neuropsychiatric disorders, making it particularly relevant to anxiety and autonomic dysfunction in PD.^54^ Interestingly, the observed differences in our data were independent of age, disease duration, or dopaminergic medication, suggesting that these findings reflect intrinsic neural mechanisms rather than disease progression or treatment effects. The disrupted relationship in PD_Anx may reflect a breakdown in the insula's ability to modulate autonomic responses, contributing to lower HRV often seen in anxious individuals.

Understanding this breakdown, including its impact on interoception, could help guide targeted interventions for managing anxiety and autonomic dysfunction in PD. Altered interoception may further exacerbate emotional and physiological imbalances, reinforcing the cycle of anxiety and dysautonomia.

We acknowledge our study limitations. The small sample size and the lack of a non‐Parkinsonian anxious control group may restrict the generalizability of our findings.

The absence of a structured diagnostic instrument (eg, Mini‐International Neuropsychiatric Interview) and a PD‐specific anxiety scale are limitations. Although classification was informed by DSM‐5 criteria, clinical judgment, and elevated HARS scores, no formal diagnostic codes or PD‐specific subtypes or triggers were assigned, which may limit diagnostic precision given the complexity of anxiety in PD.

Our neuroimaging analysis was confined to structural magnetic resonance imaging (MRI), which might miss insights into brain network and metabolic changes that precede structural alterations (such as those detectable with functional MRI) and did not allow for detailed evaluation of small nuclei like the locus coeruleus where, for example, neuromelanin‐sensitive imaging might have been informative. Finally, the lack of neuroimaging data for the HC limits our interpretation.

## Conclusions

PD patients with anxiety have lower HRV than those without anxiety or HC, likely reflecting a shift toward sympathetic predominance. Our study reveals that in PD patients, anxiety, HRV, and left insula volume are interrelated, though the cause–effect relationship remains unclear. More research is needed to explore these potential links, and longitudinal studies in at‐risk populations may help clarify the directionality of these relationships.

HRV measures provide an affordable method to evaluate the autonomic nervous system and might serve as an objective indicator of anxiety in PD. Additionally, HRV can be used as a biofeedback tool in strategies designed to reduce anxiety in PD including mindfulness practices and breathing exercises.^55^

## Author Roles

(1) Research Project: A. Conception, B. Organization, C. Execution; (2) Statistical Analysis: A. Design, B. Execution, C. Review and Critique; (3) Manuscript Preparation: A. Writing of the First Draft, B. Review and Critique.

L.R.: 1A, 1B, 1C, 2A, 2B, 3A.

A.F.: 1A, 1B, 3B.

F.P.C.: 1C.

B.I.: 1C, 2B.

I.C.: 1C.

F.B.: 2C.

M.H.: 3B.

E.P.: 1C, 2B.

F.M.: 1B, 3B.

E.M.: 1A, 1B, 2A, 2B, 3A.

## Financial Disclosures of All Authors (for the Preceding 12 Months)

L.R. served on the advisory board of Boston Scientific and Britannia, and she received speaking honoraria from Bial, Medtronic, and the International Parkinson's Disease and Movement Disorder Society. A.F. reports royalties from Springer Verlag, speaker fees and honoraria from Antag Therapeutics, American Academy of Neurology, Austrian Neurology Society, Austrian Autonomic Society, Bial, Broadview Ventures, CNSystem, Desitin, Donau Krems University, Elsevier, GE Healthcare, Healthware International, International Parkinson Disease and Movement Disorder Society, KABEG, Medizin Forum, Medtronic, Prime Global, Sanofi, and Theravance Biopharma, and research grants from the Austrian Science Fund, Medical University of Innsbruck, Mission MSA, and the Dr Johannes and Hertha Tuba Foundation. F.P.C., B.I., I.C., F.B., and E.M. do not report any conflicts of interest. E.P. receives speaking honoraria from Boston Scientific and Medtronic, and royalties from Elsevier and Oxford University Press. M.H. is a member of the Medicines and Healthcare products Regulatory Agency (MHRA) interim devices working group. F.M. received consultancy fees from Boston Scientific and Medtronic; has been a member of advisory boards of AbbVie, Boston Scientific, Merz, Medtronic, and Roche; and received speaking honoraria from AbbVie, Boston Scientific, Merz, Medtronic, Teva, and the International Parkinson's Disease and Movement Disorder Society. She has received research support from the National Institute for Health and Care Research (NIHR) and Innovate UK and receives royalties from Springer.

## Supporting information


**Table S1.** Differences in demographic and clinical data between the two Parkinson's disease (PD) populations.

## Data Availability

The data that support the findings of this study are available from the corresponding author upon reasonable request.
