# Supplementary material for: Sympathetic Shift and Insular Alteration: Unravelling the Link Between Anxiety and Heart Rate Variability in Parkinson's Disease
Source: Mov Disord. 2025 Oct 7;41(1):179–89. doi: 10.1002/mds.70069 (PMC12882058; doi:10.1002/mds.70069)
Supplement: Supplementary file 1 — Table S1. Differences in demographic and clinical data between the two Parkinson's disease (PD) populations. [file MDS-41-179-s001.docx]

**Supplementary Material**

**Supplementary Methods**

**MRI acquisition and pre-processing**

MRI acquisitions were performed in PD patients only. Patients were scanned on a separate day from the clinical assessment (approximately 4 months between assessments) using a 3.0 Tesla (3T) scanner with a 32-channel head coil (Philips dual Tx Acheiva, St George’s Hospital), while ON medications. Structural volumes were obtained using a high-resolution three-dimensional magnetisation prepared rapid gradient echo (MPRAGE) sequence (T1-weighted, TR=6.8 ms, TE=3.1ms, TI=819ms, flip angle=8°, 1mm isotropic spatial resolution). Brain extraction was performed using the FSL (FMRIB Software Library v6.0) BET tool[(Smith et al. 2004)](https://paperpile.com/c/5sHamY/b5ghu).

T1-weighted volumes from all participants were visually inspected to identify and exclude any macroscopic artifacts before preprocessing in SPM12 (Statistical Parametric Mapping, http://www.fil.ion.ucl.ac.uk/spm/) using the DARTEL algorithm. Briefly, the MR images underwent bias-field correction to address non-uniformities, followed by segmentation into grey matter (GM), white matter, and cerebrospinal fluid using a priori tissue probability maps in the International Consortium of Brain Mapping (ICBM) template space.

The individual GM images were then normalized to the Montreal Neurological Institute (MNI) template with a voxel size of 1.5×1.5×1.5 mm³ and modulated to preserve the original individual GM volumes. Lastly, all GM images were smoothed using a 8-mm full-width at half maximum (FWHM) isotropic Gaussian kernel.

**Small Volume Correction Analyses For a priori-Hypothesized Regions of Interest**

To test a-priori hypotheses, small volume correction (SVC) analyses were performed in the bilateral Insula, and basal ganglia structures including Putamen, Caudate Nucleus, Anterior Cingulate Cortex, bilateral Amygdala and Pre-Frontal Cortex. Anatomical masks for these regions were derived from the SPM-12 anatomy toolbox (Smith et al., 2004; [Eickhoff et al. 2005)](https://paperpile.com/c/5sHamY/gBFY).

### **Sample size**

A post hoc power analyses using G*Power 3.1 was conducted, focused on the primary analysis in our study: a mixed-design ANOVA examining the effects of Group (PD with anxiety, PD without anxiety, and healthy controls) and Position (sitting, standing minute 1, standing minute 3) *on logRMSSD***.**

Our main effect of interest was the Group factor, representing diagnostic status, as it directly reflects the hypothesised influence of anxiety and PD on autonomic regulation. With a total sample size of 66 participants (27 PD_Anx, 23 PD_noAnx, and 16 HC), we observed a large effect size for this factor (Cohen’s f = 0.628) and achieved >99% power to detect it at α = 0.05. Similarly, the main effect of Position also yielded a large effect size (f = 0.654), with >99% power. The Group × Position interaction was considered a secondary effect of interest, with the orthostatic challenge being used as a mean of eliciting an automatic response. It showed a smaller observed effect size (f = 0.28), corresponding to a power of 38%, suggesting that the study may have been underpowered to detect interaction effects of this magnitude.

*VBM power calculation*: Based on G*Power calculations for an independent samples t-test (α = 0.05, power = 0.80), a total of 42 participants (21 per group) would be required to detect a large effect size (Cohen’s d = 0.8) — which aligns with the statistical design employed in our group comparisons.

A post hoc power analysis was conducted using G*Power (v3.1) to estimate the statistical power for the observed group difference in grey matter volume between PD patients with and without anxiety. Based on the observed effect size (d = 1.22) at the right caudate nucleus (T = 4.21, df = 48), and group sizes of 27 and 23, the analysis yielded a power of 98.8% (α = 0.05, two-tailed).

A post hoc power analysis was performed for the group difference observed in the left insular cortex (T = 5.95, df = 48). The corresponding effect size was estimated at d = 1.72. With group sizes of 27 and 23 and α = 0.05 (two-tailed), the achieved power was >99.9%, indicating extremely high sensitivity to detect the observed effect.

**Supplementary Results**

**HR Comparison PD Off Medication vs. HC**: Position had a significant effect (p<0.001), with HR increasing from sitting to standing at minutes 1 (p<0.001) and 3 (p<0.001). The Position*Group interaction was significant (p=0.013), driven by differences between PD_Anx vs. HC (min 1: p=0.003, min 3: p=0.008) and PD_noAnx vs. HC (min 3: p=0.02).

**HR Comparison PD On Medication vs. HC**: Position had a significant effect (p<0.001), with HR increasing from sitting to standing at minutes 1 (p<0.001) and 3 (p<0.001). The Position*Group interaction was marginal (p=0.057), with no significant group differences (p=0.5).

**HR:** A significant Position effect was observed (F(2, 30)=3.457, p=0.045), with HR higher in standing vs. sitting. The Medication*Position interaction was significant (F(2, 30)=3.613, p=0.039), with HR increasing from minute 1 to minute 3 when OFF medication (p=0.02), but not when ON medication (p=0.5). Other interactions were not significant.

**Supplementary Tables**

**Supplementary Table 1: Differences in demographic and clinical data between the two PD populations.**

| \|  \| PD without anxiety  (N=27) \| PD with anxiety (N=23) \| p value \| \| \| \| --- \| --- \| --- \| --- \| --- \| --- \| \| Age \| 62.9±8.1 \| 61.8±6.9 \| \| 0.6 \| \| Biological sex \| 16/07 \| 13/14 \| \| 0.1 \| \| Disease duration \| 9.3±4.4 \| 11.1±4.4 \| \| 0.15 \| \| Total LEDD \| 829.2±443.7 \| 1088.3±376.8 \| \| **0.04** \| \| Dopamine-agonist LEDD \| 124.6±136.5 \| 152.4±169.7 \| \| 0.8 \| \| UPDRS III OFF \| 39.6±11.9 \| 45.4±12.3 \| \| 0.1 \| \| UPDRS III ON \| 22.3±10.7 \| 24.2±10.2 \| \| 0.5 \| \| SCOPA - Gastrointestinal \| 4.8±3.2 \| 6.5±2.5 \| \| 0.1 \| \| SCOPA - Urinary \| 7.2±4.2 \| 6.4±3.4 \| \| 0.5 \| \| SCOPA - Cardiovascular \| 1.3±1.9 \| 1.7±1.1 \| \| 0.1 \| \| SCOPA-Thermoregulatory \| 2±2.5 \| 3±1.4 \| \| **0.03** \| \| SCOPA - Pupillomotor \| 0.7±1.1 \| 0.9±0.7 \| \| 0.3 \| \| SCOPA - Sexual \| 1±1.2 \| 1.8±2.3 \| \| 0.5 \| \| SCOPA-AUT- Total Score \| 17.6±9.3 \| 21.1±8.4 \| \| 0.4 \| |
| --- | --- | --- | --- | --- | --- | --- | --- | --- | --- | --- | --- | --- | --- | --- | --- | --- | --- | --- | --- | --- | --- | --- | --- | --- | --- | --- | --- | --- | --- | --- | --- | --- | --- | --- | --- | --- | --- | --- | --- | --- | --- | --- | --- | --- | --- | --- | --- | --- | --- | --- | --- | --- | --- | --- | --- | --- | --- | --- | --- | --- | --- | --- | --- | --- | --- | --- | --- | --- | --- | --- | --- | --- | --- | --- | --- | --- |

Abbreviations: LEDD: levodopa equivalent daily dose, OH: orthostatic hypotension, SCOPA-AUT: The Scale for Outcomes in Parkinson's disease for Autonomic symptoms (SCOPA-AUT), UPDRS: Unified PD Rating Scale.

**Supplementary References**

[Eickhoff, Simon B., Klaas E. Stephan, Hartmut Mohlberg, Christian Grefkes, Gereon R. Fink, Katrin Amunts, and Karl Zilles. 2005. “A New SPM Toolbox for Combining Probabilistic Cytoarchitectonic Maps and Functional Imaging Data.” *NeuroImage* 25 (4): 1325–35.](http://paperpile.com/b/5sHamY/gBFY)

[Smith, Stephen M., Mark Jenkinson, Mark W. Woolrich, Christian F. Beckmann, Timothy E. J. Behrens, Heidi Johansen-Berg, Peter R. Bannister, et al. 2004. “Advances in Functional and Structural MR Image Analysis and Implementation as FSL.” *NeuroImage* 23 Suppl 1:S208–19.](http://paperpile.com/b/5sHamY/b5ghu)
